# HIV-associated neurocognitive disorder: key implications of the microbiota-gut-brain axis

**DOI:** 10.3389/fmicb.2024.1428239

**Published:** 2024-08-02

**Authors:** Aizhen Hu, Silvere D. Zaongo, Vijay Harypursat, Xin Wang, Jing Ouyang, Yaokai Chen

**Affiliations:** ^1^Clinical Research Center, Chongqing Public Health Medical Center, Chongqing, China; ^2^Department of Infectious Diseases, Chongqing Public Health Medical Center, Chongqing, China; ^3^Phase I Clinical Trial Center, Chonggang General Hospital, Chongqing, China

**Keywords:** HIV, gut microbiota, hand, neurocognitive disorder, microbiota-gutbrain axis

## Abstract

HIV-associated neurocognitive disorder (HAND) is now recognized to be relatively common in people living with HIV (PLWH), and remains a common cause of cognitive impairment. Unfortunately, the fundamental pathogenic processes underlying this specific outcome of HIV infection have not as yet been fully elucidated. With increased interest in research related to the microbiota-gut-brain axis, the gut-brain axis has been shown to play critical roles in regulating central nervous system disorders such as Alzheimer’s disease and Parkinson’s disease. PLWH are characterized by a particular affliction, referred to as gut-associated dysbiosis syndrome, which provokes an alteration in microbial composition and diversity, and of their associated metabolite composition within the gut. Interestingly, the gut microbiota has also been recognized as a key element, which both positively and negatively influences human brain health, including the functioning and development of the central nervous system (CNS). In this review, based on published evidence, we critically discuss the relevant interactions between the microbiota-gut-brain axis and the pathogenesis of HAND in the context of HIV infection. It is likely that HAND manifestation in PLWH mainly results from (i) gut-associated dysbiosis syndrome and a leaky gut on the one hand and (ii) inflammation on the other hand. In other words, the preceding features of HIV infection negatively alter the composition of the gut microbiota (microbes and their associated metabolites) and promote proinflammatory immune responses which singularly or in tandem damage neurons and/or induce inadequate neuronal signaling. Thus, HAND is fairly prevalent in PLWH. This work aims to demonstrate that in the quest to prevent and possibly treat HAND, the gut microbiota may ultimately represent a therapeutically targetable “host factor.”

## 1 Introduction

Nearly four decades after the emergence of the human immunodeficiency virus (HIV), HIV infection remains a major global public health concern (UNAIDS, 2022). As of 2022, the number of people living with HIV had reached approximately 39 million worldwide, and globally, almost 1.3 million individuals were newly infected by HIV and there were approximately 630,000 deaths in 2022 (UNAIDS, 2022). Fortunately, the development of modern antiretroviral therapy (ART) and widespread implementation thereof has significantly reined in the HIV epidemic, and has reduced its previously inevitable progression to acquired immunodeficiency syndrome (AIDS) (Raubinger et al., 2022). Despite the large number of HIV-infected individuals receiving ART globally [29.8 million (76%)] (The Global HIV and AIDS Epidemic, 2023), HIV (which is known to be a neurotropic virus), continues to affect the brain of people living with HIV (PLWH) (Wang et al., 2020). Indeed, HIV is known to have extensive effects on the neurological system (Mohamed et al., 2020), and for over a decade the term, HIV-associated neurocognitive disorder (HAND), has been used to represent a variety of neurocognitive impairments linked to HIV infection (Eggers et al., 2017).

The 2007 Frascati criteria defines HAND as an acquired cognitive impairment comprising at least two ability domains and, in severe cases, a deterioration in everyday functioning. Asymptomatic neurocognitive impairment (ANI), mild neurocognitive disorder (MND), and HIV-associated dementia (HAD) are the three stages of cognitive impairment, which are used to characterize the HAND syndrome (Antinori et al., 2007). However, in the present era of modern ART, the Frascati criteria (which were developed in 2007 to aid in the diagnosis of HAND) has been observed to overestimate HAND positivity, particularly when utilized for the diagnosis of the milder forms of the HAND spectrum. Despite this known limitation, the Frascati criteria remain applicable in contemporary times (Meyer, 2022); however, as suggested by Meyer, the criteria require revision and refinement (Meyer, 2022). Some researchers (Goodkin et al., 2014; Hakkers et al., 2018) categorize HAND into two forms, namely mild (ANI and MND) and severe, instead of the three forms presented in the 2007 classification. Other diagnostic tools exist, *viz.*, the international HIV dementia scale (IHDS) and the Montreal cognitive assessment-basic (MoCA-B), and others. It should also be noted here that, similar to the Frascati criteria, the preceding diagnostic methods also possess inadequate psychometric parameter assessment capacity, further limiting their diagnostic accuracy (Mwangala et al., 2018). Although severe neurocognitive impairment has become rare since the introduction of modern highly active antiretroviral therapy (HAART), HAND manifestations, identified by cognitive impairment in HIV-1 infected individuals, persist (Wang et al., 2020). Contemporarily, HAND remains a common cause of cognitive impairment worldwide (Saylor et al., 2016). At the same time, other potential contributing factors for the occurrence of HAND have now been described, including persistent latent HIV-1 reservoirs in the brain, irreversible central nervous system (CNS) insult prior to ART initiation, toxicity associated with antiretroviral drugs, host genetic factors predisposing to the emergence of HAND, deposition of amyloid and tubulin associated unit (Tau) protein, neuroinflammation, as well as damage (at a molecular level) to various neurotransmitter systems (Chang et al., 2008; Nagano-Saito et al., 2009; Hammoud et al., 2010; Sperner-Unterweger et al., 2014; Keegan et al., 2016).

Recently published investigations have highlighted the impact of gut microbiota on the gut-brain axis, and their potential influence on CNS-related diseases and neuropsychiatric disorders. Given that the gut microbiota and their microbial metabolites have been shown to profoundly affect host immunity, cognition, behavior, and metabolism (Marques et al., 2010; Cryan and O’Mahony, 2011; Foster and McVey Neufeld, 2013), increasing attention has been focused on the potential implications of the gut microbiota and microbial metabolites with respect to HAND pathogenesis. The preceding factors suggest that the microbiota-gut-brain axis may represent a thus far unrecognized therapeutic target in the quest to treat HAND. This therapeutic approach may potentially be highly beneficial, as HAND (i) induces a psychological and emotional burden on families, friends, and relatives of HIV positive patients, (ii) provokes difficulties with medication (ART) compliance and follow-up (Sharma, 2021; Zenebe et al., 2021), and (iii) causes HIV positive individuals to engage in high-risk behavior (such as casual unprotected sexual intercourse) favoring HIV transmission (Shrestha and Copenhaver, 2016). Furthermore, the emergence of HAND in HIV-infected individuals places a further burden on already strained resources in hospitals and medical facilities, and also exerts a financial toll on national health resources, even in affluent nations. Therefore, a comprehensive investigation into the role of the microbiota-gut-brain axis in HAND pathogenesis, and also in the treatment and prevention of HAND, may also pave the way toward more robust strategies against HIV infection.

In this review, we comprehensively discuss and highlight the role of the microbiota-gut-brain axis in modulating enteric and central nervous system functions from a clinical perspective. Specifically, we (i) briefly review the prevalence of neurological disorders in HIV-infected individuals, (ii) extensively discuss the mechanisms whereby gut integrity influences the onset of neurological disorders, and (iii) discuss the influence of HIV-associated gut dysbiosis in the onset of neurological outcomes via the consequences of dysbiosis on alterations in the microbiota-gut-brain axis.

## 2 High neurological disorder prevalence in PLWH

Prior to the present era of modern combination antiretroviral therapy, severe cognitive impairments were reported in up to 50% of people living with HIV (Grant et al., 1987). Although severe neurocognitive impairment secondary to HIV infection has become rare as a result of successful immune reconstitution in this era of modern and potent antiretroviral therapy, milder forms of cognitive impairment remain common in PLWH (Heaton et al., 2010; Clifford and Ances, 2013). To illustrate this point, observations from one recent meta-analysis (of 123 studies conducted in 32 countries) have indicated that the overall prevalence of HAND in HIV-infected adults was calculated to be 42.6%, equating to roughly 16,145,400 cases worldwide. Specifically, the prevalence of asymptomatic neurocognitive impairment (ANI), mild neurocognitive disorder (MND), and HIV-associated dementia (HAD) were observed to be 23.5, 13.3, and 5.0%, respectively (Wang et al., 2020). Additionally, longitudinal cohort observations have revealed that the presence of ANI, when compared against neurocognitively normal individuals, confers a 2–6-fold increase in risk for earlier development of symptomatic HAND (Grant et al., 2014). These observations are valid even in ART-treated individuals with undetectable HIV viral loads (Grant et al., 2014). Moreover, several observational cohort studies have observed that PLWH on antiretroviral therapy (ART) are relatively more likely to develop dementia compared to people without HIV (Lam et al., 2021, 2022). One of the preceding cohort studies observed that compared to HIV negative individuals, PLWH have a 58% higher risk of developing dementia (Lam et al., 2021). Interestingly, exposure to ART did not reduce this risk. Notably, major depressive disorder (MDD) is the most prevalent psychiatric manifestation associated with HIV infection (Dubé et al., 2005), with a prevalence of up to 36% (Gaynes et al., 2015; Passchier et al., 2018), which is at least twice that observed in healthy community samples (Chibanda et al., 2014; Passchier et al., 2018). Overall, HIV is strongly implicated in the onset of HAND in ART-treated or ART-naïve PLWH. HIV neurotropism in the brain, which is characterized by inflammation resulting from HIV viral protein interactions with endothelial cells (Younas et al., 2016), increased production of reactive oxygen species (ROS) (Cirino et al., 2022), and brain damage resulting from the destruction of astrocytes and pericytes (Ahmed et al., 2018), is possibly responsible for the development of HAND. However, the potential etiological factors for HAND development, which relies solely on the preceding components will be an incomplete description of all the likely causal factors. We believe that the gut may well be seen as a further protagonist in the pathophysiological evolution of HAND. Thus, a picture depicting the influence of HIV on the gut is worth exploring.

## 3 HIV infection is associated with gut microbiota dysbiosis and related inflammation

The gut microbiota is composed of a community of microorganisms contained within the gastrointestinal (GI) tract, and exists symbiotically with the human host (Thursby and Juge, 2017). A healthy and stable gut microbiota community plays a vital role in maintenance of homeostatic balance of gut barrier integrity, gut function, gut metabolism, and immunity of the gut (Sun and Shen, 2018). It is now well established that profound changes (in microbial composition, metabolites, and immune cells) occur within the gut of an HIV-infected individual.

The GI tract is known to harbor the majority of the body’s complement of immune cells (de Waele, 2021). The vast population of activated memory CD4+ T-cells, with abundant expression of chemokine receptors, provides HIV-1 with an ideal environment to establish infection (Mehandru et al., 2005). Indeed, activated CD4+ T-cells within the gut, in addition to predominantly expressing CXCR4 and CCR5 receptors [which facilitates HIV penetration of these cells (Poles et al., 2001)], are one of the primary targets of HIV (Zaongo et al., 2022). As such, researchers have hypothesized that interventions aimed at reducing the vulnerability of such cells may lower the risk of HIV acquisition (Lajoie et al., 2021). Thus, HIV infection can lead to rapid and substantial depletion of CD4+ T-cells in the lamina propria (Ishizaka et al., 2021). This depletion of CD4+ T-cells is mediated predominantly by apoptosis (Cooper et al., 2013), pyroptosis (Doitsh et al., 2010), and cytotoxic T-cells (Chávez-Galán et al., 2009; Liu et al., 2011). HIV also directly attacks the gut mucosal epithelium, resulting in intercellular tight junction disruption, death of enterocytes, and ultimately a more permeable gut (Younas et al., 2019). The preceding scenario “opens the gate,” so to speak, to gut-associated dysbiosis syndrome, displacement of microbial product into the bloodstream (microbial translocation), and systemic inflammation (Younas et al., 2019). Moreover, researchers have reported that CD4+ T-cell depletion occurs at all stages of HIV disease, and predominantly occurs in the GI tract (Brenchley et al., 2004). Although ART may restore CD4+ T-cells in other anatomical locations, lymphocyte levels within the gut, in the majority of cases, are slow to return to normal levels and restoration of their numbers is most often incomplete (Kotler, 2005). Gut microbiota dysbiosis has the potential to influence HIV disease in various ways throughout all phases in the natural history of HIV disease progression, from transmission to end stage disease (Li et al., 2016).

According to previous research data, gut microbiota dysbiosis in PLWH mainly manifests as alterations in microbial diversity and relative abundance of certain specific gut microorganisms (Dillon et al., 2016; Deusch et al., 2018). The diversity of gut microbiota in HIV-infected people is significantly lower than that of the general population (Lu et al., 2018). One recent meta-analysis examined 22 studies to evaluate alpha (α-) diversity in the gut microbiota of HIV-infected compared to HIV-uninfected individuals, and concluded that HIV status was associated with a decrease in measures of α-diversity (Tuddenham et al., 2020). Apart from changes in diversity, HIV infection is associated with depletion of commensal species and enrichment of opportunistic pathogens (McHardy et al., 2013). Several survey studies in human cohorts have compared the intestinal microbiota composition of HIV-positive patients with that of HIV-uninfected individuals (Lozupone et al., 2013; McHardy et al., 2013; Vujkovic-Cvijin et al., 2013; Dillon et al., 2014; Mutlu et al., 2014; Yu et al., 2014; Dinh et al., 2015; Nowak et al., 2015; Vázquez-Castellanos et al., 2015; Dubourg et al., 2016; Ling et al., 2016; Monaco et al., 2016; Noguera-Julian et al., 2016; Sun et al., 2016; Vesterbacka et al., 2017; Armstrong et al., 2018; Lee et al., 2018; Lu et al., 2018; San-Juan-Vergara et al., 2018; Zhou et al., 2018; Vujkovic-Cvijin and Somsouk, 2019), and these have shown an enrichment of *Erysipelotrichaceae*, *Enterobacteriaceae*, *Desulfovibrionaceae*, and *Fusobacteria*, and a reduction of *Lachnospiraceae* (Vujkovic-Cvijin and Somsouk, 2019). *Ruminococcaceae* and *Lachnospiraceae* taxa comprise the primary producers of short-chain fatty acids (SCFA) within the gut, meaning that their relative depletion in HIV-infected individuals is also associated with a diminution of SCFA in the gut. Moreover, in HIV-infected individuals, a proliferation of proinflammatory microorganisms such as *Candida albicans*, and a diminution of anti-inflammatory microorganisms such as *Akkermansia muciniphila*, is observed (Ouyang et al., 2020; MacCann et al., 2023).

In addition to microbial composition, HIV infection may also cause dysregulation of gut microbiota metabolism (McHardy et al., 2013). SCFAs, which are the fermentation products of intestinal microbiota, is crucial for maintenance of the overall health of intestinal epithelial cells and regulation of local immune responses (Shi et al., 2017). Compared with HIV-negative individuals, circulating levels of butyric acid and valeric acid are reduced in HIV-positive patients (Qing et al., 2019). Moreover, levels of butyric and valeric acids positively correlate with the abundance of species such as *Rikenellaceae*, *Ruminococcaceae*, *Alistipes*, *Roseburia*, and *Lachnospiraceae* (Qing et al., 2019). Interestingly, different metabolic functions manifest based on the different microbial communities that are more abundant in HIV-infected subjects. McHardy et al., have observed that imputed metagenomic functions, including amino acid metabolism, vitamin biosynthesis, and siderophore biosynthesis differs significantly between healthy controls and HIV-infected subjects not receiving ART (Kang and Cai, 2019). Gut-resident bacteria with the capacity to metabolize tryptophan (TRP) through the kynurenine (KYN) pathway were observed to be enriched in HIV-infected subjects (Vujkovic-Cvijin et al., 2013), and gut microbiota communities in HIV-infected subjects exhibit an increased capacity to catabolize tryptophan to kynurenine (Serrano-Villar et al., 2016; Vujkovic-Cvijin and Somsouk, 2019). Degradation of TRP via the KYN pathway may result in decreased production of serotonin. Interestingly, several studies have noted low levels of serotonin [5-hydroxytryptamine (5-HT)] in the blood and CSF of patients with HIV-1 infection (Launay et al., 1988; Larsson et al., 1989; Ghare et al., 2022).

Thus, HIV infection is associated with alterations in microbiota composition and microbial metabolites, and physical disruption of the gut endothelial barrier. These changes may contribute to greater microbial translocation and the persistence of a proinflammatory state even subsequent to restoration of circulating CD4+ T-cell counts via ART (Takiishi et al., 2017; MacCann et al., 2023). The preceding repercussions of HIV infection within the gut, together with their consequences, may well influence the gut-brain axis and possibly mediate the subsequent development of HAND.

## 4 Neurocognitive disorders and their connections to gut microbiota as reported in HIV negative contexts

Cumulative research evidence implicates the gut microbiosis in a variety of psychiatric, neurological, and neurodegenerative diseases (Cryan et al., 2019). Evidence from both clinical and experimental studies show that there is an imbalance of gut microbiota and microbial metabolites present in various CNS diseases (Table 1). For example, clinical studies on the gut microbiota of patients with Alzheimer’s Disease (AD) and the gut microbiota in an AD mouse model have suggested differences in microbial diversity, compared to the control group. Specifically, it has been observed that AD is associated with a decrease in *Fusobacteriaceae*, *Firmicutes*, *Actinobacteria*, and *Bifidobacterium*, and an increase in *Bacteroidetes* (Westfall et al., 2017; Megur et al., 2020). In fecal samples from Parkinson’s disease (PD) patients, bacteria more commonly related to anti-inflammatory properties, such as genus *Blautia*, *Coprococcus*, and *Roseburia*, are significantly decreased, while proinflammatory *Proteus*, *Enterococcaceae*, and *Enterobacteriaceae* organisms increased (Keshavarzian et al., 2015; Scheperjans et al., 2015; Bedarf et al., 2017; Quigley, 2017). Similarly, recent studies (Jiang et al., 2015; Aizawa et al., 2016; Sun and Shen, 2018) have suggested that (i) lower *Bifidobacterium* and *Lactobacillus* levels are more common in individuals with major depressive disorder, (ii) *Faecalibacterium* levels are negatively associated with severity of depressive symptoms in patients with major depressive disorder, and (iii) *Enterobacteriaceae* and *Alistipes* proportions are increased in people having major depressive disorder, compared to healthy controls. Recently, it has been reported that gut microbiota from patients with HAND showed significantly lower α-diversity compared with that from patients without HAND (Sun et al., 2016; Hamad et al., 2018; Zhang et al., 2018). Notably, the gut microbiota composition in HIV positive patients with neurocognitive impairment is significantly different from those without neurocognitive impairment (Dong et al., 2021). HIV positive individuals with neurocognitive impairment also present a decreased abundance of butyrate-producing bacteria (BPB) and an increased abundance of *Klebsiella* (Dong et al., 2021).

**Table 1:** Examples of altered gut microbiota composition in HIV-negative patients with CNS diseases or neurocognitive disorders.

Diseases	Altered gut microbiota	Subjects	References
Alzheimer’s disease	*Bacteroidetes*↑, *Fusobacteriaceae*↓, *Firmicutes*↓, *Actinobacteria*↓, and *Bifidobacterium*↓	Humans, rats	Salminen et al. (1998); Westfall et al. (2017); Megur et al. (2020)
Parkinson’s disease	*Proteus proinflammatory*↑, *Enterococcaceae*↑, *Enterobacteriaceae*↑, *genus Blautia*↓, *Coprococcus*↓, and *Roseburia*↓	Humans	Keshavarzian et al. (2015); Scheperjans et al. (2015); Bedarf et al. (2017); Quigley (2017)
Major depressive disorder	*Enterobacteriaceae*↑, *Alistipes*↑, *Bifidobacterium*↓, *Lactobacillus*↓, and *Faecalibacterium* ↓	Humans	Jiang et al. (2015); Aizawa et al. (2016); Sun and Shen (2018)

↑: augmentation; ↓: reduction.

Interventions utilizing therapeutic modalities such as antibiotics, probiotics, and fecal microbiota transplantation (FMT) may modify the composition of the gut microbiota and therefore influence the cognitive functioning of the host. Indeed, antibiotics are known to disrupt the gut microbial community, which may have negative consequences on brain function and behavior. In rodents, antibiotic administration induces changes in the gut microbiota, and this has been observed to be associated with subsequent object recognition memory impairment and altered hippocampal function (Desbonnet et al., 2015; Fröhlich et al., 2016; Möhle et al., 2016; Cryan et al., 2019). Additionally, through fecal microbiota transplantation (FMT), it has been demonstrated that transplanted gut microbiota may improve the symptoms of neurological disease by modulating the microbiota-gut-brain axis. For instance, one murine model of AD observed that microbiota transplantation utilizing the gut microbiota from a healthy subject alleviates the formation of amyloid beta (β) plaques and neurofibrillary tangles, as well as improves glial reactivity, and cognitive impairment (Kim et al., 2020). Thus, via the observations gleaned from FMT experiments, it can be seen that gut microbiota may be able to transfer a behavioral phenotype or disease feature to a recipient, providing stronger evidence for a causal relationship between gut microbiota and CNS disease. The utilization of specific probiotics in humans has also demonstrated beneficial effects with respect to cognitive performance in both healthy and unhealthy individuals (Cryan et al., 2019). Significant improvements in various cognitive test scores (inclusive of memory, attention, executive function, and language) were observed in two studies in which PLWH received probiotics as supplements (Ceccarelli et al., 2017). This indicates that the gut-brain axis plays a vital role in the manifestation of neurological disorders. Furthermore, in a recent publication, our team (Zaongo et al., 2023) has published extensive information regarding the elements of the gut, which may influence brain development and functioning. We believe that the gut microbiome may also play critical roles in the development of neurodegenerative disorders (Table 2), inspired from our published article (Zaongo et al., 2023). Notably, according to previously published investigations (Morais et al., 2021; Mayer et al., 2022), it is hypothesized that the specific pathways which potentially define the relationships between the gut-brain axis and neurological disorders are either direct (direct effects on neurons and neuronal signaling), or indirect (via immune responses and/or microbial metabolites).

**Table 2:** Elements of the gut which are potentially involved in the development of neurodegenerative disorders.

Groups	Specific elements	Utility/reported effect	References
Metabolites or neurotransmitters	Serotonin	At the pre-natal level, is essential for fetal forebrain development.	Bonnin et al. (2011)
	Vitamins K2 and B12	Essential for human survival and nervous system development.	Dror and Allen (2008); Santos et al. (2008); Marques et al. (2010)
	Acetate	Regulates microglial functions.	Erny et al. (2021)
	Butyrate	Influences brain functions as it may regulate gene expression in the brain.	Bourassa et al. (2016) Alpino et al. (2022)
	Kynurenines (kynurenic acid, 3-hydroxykynurenine, quinolinic acid, and 3-hydroxyanthranilate)	They target neurotransmitter receptors and affect redox processes, and thus influence brain physiology.	Schwarcz et al. (2012)
	Lipopolysaccharides	Induces anxiety or depressive-like or sickness behavior (fatigue, anorexia, low mood, or apathy later in life).	DellaGioia and Hannestad (2010); On Wah et al. (2019)
Bacteria	*Lactobacillus reuteri*	Produces vitamin B12, which is essential for nervous system development	Dror and Allen (2008); Marques et al. (2010)
	*Clostridium aminobutyricum*	Responsible for infections located in the gut. These infections are linked to autism and schizophrenia as they influence the developmental programming of the brain.	Finegold et al. (2002); Finegold (2008); Mittal et al. (2008); Bale et al. (2010)
	*C. bifermentans*		
	*C. clostridioforme*		
	*C. difficile*		
	*C. Colceatum*		
	*C. nexile*		
	*C. orbiscindens*		
	*C. ramosum*		
	*C. roseum*		
	*C. scindens*		
	*Campylobacter jejuni*	Induces behavioral abnormalities including anxiety and impaired cognition.	Bilbo et al. (2005); Sullivan et al. (2006); Goehler et al. (2008)
	*Escherichia coli*	Induces behavioral abnormalities including anxiety and impaired cognition	Bilbo et al. (2005); Sullivan et al. (2006); Goehler et al. (2008)
	Bifidobacteria	May influence brain fatty acid composition	Wall et al. (2012)
		In probiotics (predominantly *Bifidobacterium longum* and *Bifidobacterium bifidum*), they are suspected to have beneficial effects on depression in rats exposed to maternal separation stress in early life	Shkoporov et al. (2008)
	*Slackia*	Produces equol, which is essential in maintaining homeostasis.	Schröder et al. (2013); Freedman et al. (2018)
	*A. muciniphila*	May produce serotonin.	Valles-Colomer et al. (2019)
		Produces acetate, which has beneficial effects on neurodegenerative conditions.	Mirzaei et al. (2021)
		Produces propionate, which has beneficial effects on neurodegenerative conditions.	Mirzaei et al. (2021)
		Reduces inflammation by producing indole and indole acetic acid from tryptophan metabolism.	Schröder et al. (2013); Freedman et al. (2018); Liu et al. (2020)
	*Eubacterium hallii group*	Produces propionate, which has beneficial effects on neurodegenerative conditions.	Durack and Lynch (2019)
		Produces butyrate.	Liu et al. (2020)
	*Lactococcus*	Influences levels of serotonin (modulates serotonin signaling/metabolism).	Oluwagbemigun et al. (2022)
		Produces histamine, which is essential to regulate cognitive functions.	Landete et al. (2008); Thomas et al. (2012)
		Production of dopamine, which is essential to regulate cognitive functions.	Tetz et al. (2018)
	*Pseudomonas*	Influences levels of serotonin (modulates serotonin signaling/metabolism).	Oluwagbemigun et al. (2022)
		Production of gamma aminobutyric acid, a marker of Alzheimer’s disease.	Manyevitch et al. (2018); Strandwitz et al. (2019)
Fungi	*Candida albicans*	Memory impairment.	Wu et al. (2019)
	*Geotrichum capitatum*	Can disseminate in lung, liver, and skin. Thus, it may potentially infect the brain and influence its functioning.	Gao et al. (2015)
	*Saccharomyces boulardii*	Improves the behavior and emotions.	Tao et al. (2021)
Virus	HIV	HIV induces neuronal apoptosis.	Ozdener (2005); Soontornniyomkij (2017)
		Induces permanent gut dysbiosis syndrome, which allows translocation of microbial products in blood. Once in contact with the brain, they influence its functioning.	van Marle et al. (2013); Rich et al. (2020)
		Could influence brain function by killing enteroendocrine cells within the gut.	
		Chronic inflammation whereby cytokines produced by immune cells can influence the brain functioning.	Maek et al. (2007); Benakis et al. (2016); McArthur and Johnson (2020); Salvo-Romero et al. (2020)
Enteroendocrine cells	EC cells, D cells, I cells, K cells, and L cells	Produces neuroactive molecules (5-hydroxytryptamine/serotonin) and peptides (cholecystokinin, peptide YY, and glucagon-like peptide 1), which modulate brain functions via enteric nervous system, vagus nerve, and spinal afferent fibers.	Latorre et al. (2016); Needham et al. (2020)
Immune cells (CD4+ T-cells, dendritic cells, macrophages, etc.)	Pro-inflammatory cytokines (IL-1, IL-17, and IFN gamma)	Modulates brain development and functions via their receptors located in the hippocampus.	Salvo-Romero et al. (2020)

### 4.1 Neurons and neuronal signaling

Two neuronal pathways physically link the gut and the brain (Carabotti et al., 2015). These comprise the direct connections via the vagus nerve between the brain and the gut and the bidirectional exchange between the brain and the gut via the enteric nervous system (ENS) present in the intestine (Gwak and Chang, 2021). The vagus nerve is a significant component of both pathways. Indeed, the vagus nerve (VN) extends from the brainstem as the tenth (and longest) cranial nerve, to innervate the gut and the enteric nervous system (ENS) (Breit et al., 2018). Interestingly, Yoo and Mazmanian have observed that intestinal microbiota may mediate the development and the functional components of the enteric nervous system (ENS) (Yoo and Mazmanian, 2017). Similarly, De Vadder et al. (2018), have demonstrated that neuronal innervation of colonic epithelium is reduced in germ-free (GF) mice, and that this phenotype is reversed 15 days after colonization by gut microbes. Microbes and their products regulate the development and maturation of glial cell networks within the nervous system, and enteric glial cells (EGCs) are the major target of the gut microbiota (Kabouridis et al., 2015). Furthermore, gut microbiota may affect the function of enteric neurons through chemical signaling. Indeed, it has been reported that supplementation with SCFA-producing gut microorganisms may suppress the activation of the neuronal pathway that mediates gut motility (Morais et al., 2021). Additionally, direct neural communication between gut microbiota and the brain is mainly achieved through the VN (Borre et al., 2014). As such, the VN sensory fibers innervate the muscle and mucosal layers of the gastrointestinal tract, detect sensory signals, and subsequently transmit these signals to the CNS (De Vadder et al., 2018). In animal studies, injection of α-synuclein (a neuronal protein that regulates synaptic vesicle trafficking and subsequent neurotransmitter release) into the duodenal and pyloric muscularis layer induces its dissemination along the VN to the middle brain, causing neuronal damage (Kim et al., 2019; Van Den Berge et al., 2019; Benakis et al., 2020). Although the VN is likely to be involved in the pathogenesis of both acute and chronic brain disorders, its role in linking the gut microbiota to the natural history of any particular disease has not as yet been extensively investigated (Benakis et al., 2020).

### 4.2 Modulation of immune responses

The gut microbiota interacts intimately with the intestinal immune system. Microbial interactions with local immune cells may lead to functional changes that extend beyond the gastrointestinal tract. According to Agirman et al., this scenario may occur through alterations to the release of cytokines into the systemic circulation or through conditioning of immune cells that home to other anatomical sites, including the brain (Agirman and Hsiao, 2021). Notably, proinflammatory cytokines (IL-1, IL-17, and IFN-γ) have been observed to be capable of modulating brain development and functions via their receptors located in the hippocampus (Tetz et al., 2018). In addition to microbial effects on peripheral immune cells, the microbiota is also necessary for healthy development, maturation, and activation of microglia, which are the innate immune cells of the brain (Abdel-Haq et al., 2019; Morais et al., 2021). Moreover, it has been reported that germ-free (GF) mice display global defects in microglia, with altered cell proportions and an immature phenotype, leading to impaired innate immune responses within the murine brain (Erny et al., 2015). Defective microglia are also associated with limited microbiota complexity. Conversely, recolonization of the gut with a complex gut microbiota (particularly enriched with SCFA and bacteria producing SCFA) partially restores normal microglia phenotypic traits (Erny et al., 2015). A separate research group has also demonstrated that short-chain fatty acids and microbiota-derived bacterial fermentation products may restore microglial morphology and function (Erny et al., 2015). Crucially, alterations in microglial function have been linked to stress, behavioral disorders, and neurodegenerative disorders, which suggests that the gut microbiota may influence human neurological diseases through effects mediated by microglia (Morais et al., 2021). The permeability of the blood–brain barrier has been shown to be related to the gut microbiota and microbial metabolites. Some reports show that GF mice have increased blood–brain barrier (BBB) permeability relative to control mice (Braniste et al., 2014). The increased permeability of the gut and the blood–brain barrier induced by gut microbiota derangement may thus potentially mediate or influence the emergence of neurodegenerative disorders (Jiang et al., 2017).

### 4.3 Microbial metabolites

Microbial products and metabolites, including secondary bile acids, indole-derivatives, and SCFAs may transmit signals through enteroendocrine cells (EECs) and enterochromaffin cells (ECCs) to regulate the secretion of neuropeptides and neuromodulators such as the hormonal neurotransmitter, serotonin. Furthermore, subsets of gut bacteria may directly synthesize and release specific neurotransmitters and neuromodulators (Agirman and Hsiao, 2021). For example, *Enterococcus* spp., *Escherichia* spp., *Lactobacillus* spp., *Lactococcus* spp., *M. morganii*, and *Streptococcus* spp., to list a few, have been observed to produce serotonin (Agirman and Hsiao, 2021).

#### 4.3.1 Short chain fatty acids

Short-chain fatty acids improve gut motility, reduce the release of proinflammatory cytokines, and modulate adaptive immune tolerance as well as the levels of gut hormones and neuropeptides (Dalile et al., 2019; Benakis et al., 2020). SCFAs directly impact brain function, regulating BBB permeability, microglial function, and modulation of neuroinflammatory responses. As an example, Erny et al., have demonstrated that mono-colonization by a butyrate-producing bacterium restores the integrity of the BBB in GF mice, and also plays a critical role in the maturation of microglia (Erny et al., 2015). In addition, SCFAs may affect neuro-inflammation by modulating the production and recruitment of immune cells such as T-cells and neutrophils, and of inflammatory cytokines (Parker et al., 2020). Butyrate has been reported to stimulate memory and synaptic plasticity by inhibition of histone deacetylases (Kaur et al., 2019). SCFAs may also influence health and behavior. For instance, mice exposed to acute exogenous SCFA (sodium butyrate) are observed to have altered production of brain-derived neurotrophic factor (BDNF), which is a neuronal factor that has been associated with depression (Schroeder et al., 2007). In the preceding study, prolonged injection of exogenous sodium butyrate into mice (for 28 days) has been observed to reduce their depressive-like behaviors in a statistically significant manner.

#### 4.3.2 Serotonin

The gut microbiota influences tryptophan (TPH) metabolites, and thus affects the pathogenesis of many neurologic and psychiatric disorders. TPH is the only substrate for serotonin synthesis, which occurs primarily in the distal gastrointestinal tract (90%) and, to a lesser extent, in the central nervous system (10%) (Roth et al., 2021). The commensal gut microbiota has multiple regulatory mechanisms for the peripheral serotonin pool. On the one hand, microbial metabolites such as indole, SCFAs, and secondary bile acids impact the generation and secretion of 5-HT by enteroendocrine cells (EECs) (Gao et al., 2020; Agirman and Hsiao, 2021). On the other hand, the commensal gut microbiota may directly utilize tryptophan to synthesize serotonin. Indeed, it has been reported that specific bacterial strains, such as *Lactococcus*, *Lactobacillus*, *Streptococcus*, *Escherichia coli*, and several bacteria of the *Klebsiella* genus, may produce serotonin by expressing tryptophan synthase (O’Mahony et al., 2015; Gao et al., 2020). Apart from changes to the peripheral serotonin pool, modulation of central serotonin metabolism by gut microbiota also occurs in multiple ways. Observations from some studies (Gao et al., 2018, 2019; Lukić et al., 2019) imply that alterations to gut microbial tryptophan metabolism may influence changes in central serotonin metabolism by affecting tryptophan availability. Moreover, the gut microbiota exhibits other pathways, which modulate central serotonin synthesis (Gao et al., 2018, 2019; Lukić et al., 2019). For example, some microbial metabolites (especially butyrate, which may be transported into the systemic circulation) are thought to have neuroprotective effects in stressed mice via an increase in brain serotonin levels and a remediation of BBB impairments (Sun et al., 2016). Additionally, inflammatory stimuli have been observed to decrease serotonin levels in the prefrontal cortices of mice (Zhu et al., 2015). In the CNS, 5-HT is involved in the modulation of a range of mood, behavioral, and cognitive functions (Cryan and Leonard, 2000; Berger et al., 2009; Kennedy et al., 2017), and low serotonin levels have been reported to be associated with depression, fatigue, and impaired cognitive functions (Geldenhuys and Van der Schyf, 2011; Kaur et al., 2019).

#### 4.3.3 Tryptophan metabolism

Approximately 90% of tryptophan is metabolized along the kynurenine pathway. TRP can be catabolized by the heme-dependent enzymes, TRP 2,3-dioxygenase (TDO) and indoleamine 2,3-dioxygenase (IDO1), resulting in the production of kynurenine (KYN) and its derivatives (Stone, 1993; Guillemin et al., 2003; Richard et al., 2009). Fluctuating levels of kynurenine pathway metabolites, including kynurenine, kynurenic acid, 3-hydroxyanthranilic acid (3-HAA), 3-hydroxykynurenine (3-HK), and the neurotoxic quinolinic acid are associated with many neurologic and psychiatric disorders (Schwarcz et al., 2012; Kennedy et al., 2017). Kynurenine and quinolinate, for instance, have been proposed as metabolites which are likely to perturb brain functioning and consequently cause depression-like symptoms (Oxenkrug, 2013; Kaur et al., 2019). The preceding observations indicate that the gut microbiota may influence brain functions through modulation of the kynurenine pathway. Studies have reported that the gut microbiota not only regulates the expression of the kynurenine pathway genes in the hippocampus via an microRNA-dependent mechanism, but may also modulate the kynurenine pathway in the brain by directly impacting the activity of its key enzymes (Malmevik et al., 2016; Moloney et al., 2017; Gao et al., 2020). Additionally, circulating SCFAs, such as butyrate, may directly modulate central kynurenine pathways (Agirman et al., 2021; Sanmarco et al., 2021). Notably, the kynurenine pathway plays a primary role in influencing tryptophan availability by the clearance of excess tryptophan. Thus, dysregulation between serotonin synthesis and the kynurenine pathway may incite the emergence of neuropsychiatric disorders, such as depression (Kennedy et al., 2017; Gao et al., 2020). Other than serotonin synthesis and the kynurenine pathway, gut microbiota may directly transform tryptophan into indole derivatives. Subsequently, indoles of bacterial origin may be incorporated into the systemic circulation, may cross the BBB, and may exert neuroprotective effects via aryl hydrocarbon receptor (AhR) signaling (Rothhammer et al., 2016). One recent study observed that the microbial indole metabolites of tryptophan, including indole, indole-3-acetic acid (IAA), and indolic-3-propionic acid (IPA), may activate AhR signaling in astrocytes and hence modulate CNS inflammation (Rothhammer et al., 2016).

#### 4.3.4 Membrane-derived molecules

Other gut microbiota-derived molecules may have significant effects on host immunity and neurological diseases (Benakis et al., 2020). A prominent example is the endotoxin, lipopolysaccharide (LPS). LPS translocation is facilitated by gut permeability, which also causes a potent inflammatory response that may damage/disrupt the BBB and subsequently activate microglia (Banks and Erickson, 2010). For instance, *Proteus mirabilis* gavage has been observed to replicate PD-like symptoms, and enhances microglia activation via LPS in wild-type mice (Choi et al., 2018).

Three parallel but related communication pathways may be used to send inflammatory signals from the GI tract to the central nervous system (Agirman et al., 2021). Intestinal inflammation (triggered by dysbiosis) induces the release of proinflammatory cytokines and may have dramatic extraintestinal consequences (Parker et al., 2020). Circulating proinflammatory factors may disrupt epithelial tight junctions and compromise both the gut-vascular barrier (GVB) and BBB integrity (Parker et al., 2020), thus “opening the door” for molecules, toxins, and pathogens originating from the gut lumen to enter the brain parenchyma, activating local immune cells, and inducing neuroinflammation. Increased systemic levels of the bacterial wall component, LPS, for instance, has been linked to cognitive decline, microglial activation, neuronal cell death, and cytokine-mediated illness behavior (Zhao et al., 2019). Moreover, the gut microbiota and their byproducts have been shown to have a direct impact on neuroimmunomodulatory functions within the CNS (Agirman et al., 2021), and a lack of immune priming results in an inadequate response to brain insults and inflammatory stimuli (Erny et al., 2015; Rothhammer et al., 2016; Thion et al., 2018; Van Hove et al., 2019). Local immune cells of the CNS may also be programmed by gut-derived cells whose functions can be regulated by the gut microbiota (Agirman et al., 2021; Sanmarco et al., 2021). In the case of gut dysbiosis, intestinal immune cells may directly promote CNS neuroinflammation (Agirman et al., 2021). Multiple sclerosis (MS) is the most prevalent form of inflammatory disease in the CNS, and mounting evidence suggests that other neurological diseases such as AD, PD, and autism spectrum disorder (ASD) may also be significantly associated with inflammatory responses (Heneka et al., 2015; Park and Kim, 2021). Although each neurodegenerative disease has its own unique pathway that ultimately results in neurodegenerative changes, chronic inflammation that originates from and is dependent on the gut microbiota is often a key aspect of the progressive nature of neurodegeneration (Harry, 2013). Immune reactions in the CNS may reportedly have adverse long-term effects, especially in cases of chronic inflammation, and proinflammatory cytokines and oxidative stress have been causally linked to neuronal death (Millán Solano et al., 2023).

From the preceding details, it is fair to reason that the crosstalk between the gut and the brain is a crucial factor to consider when contemplating the fundamental nature of neurocognitive disorders, and this gut-brain crosstalk may involve multiple mechanisms. As such, and based on current evidence, we believe that the gut-brain axis plays a significant role in the pathogenesis of neurocognitive disorders that are encountered in PLWH.

## 5 The microbiota-gut-brain axis mediates neurological disorders in PLWH: evidence from the literature

Evidence from contemporary literature informs that HIV infection may affect both the brain and the gut. It is known that HIV may overcome BBB protection and penetrate the CNS [via a “trojan horse” (i.e., an infected CD4+ T-cell or a monocyte migrating from the bloodstream into the CNS), or via transcytosis (i.e., infected epithelial cells transporting HIV particles from the systemic circulation into the CNS)], where it may infect different types of cells (macrophages, microglia, and astrocytes), promote inflammation, and provoke neuronal damage (Araínga et al., 2017; Abreu et al., 2019; Wallet et al., 2019; McArthur and Johnson, 2020). The mechanisms (involving neuroinflammatory responses to viral proteins and inflammatory cytokines released by infected microglia and macrophages) whereby HIV exerts deleterious effects on neurons are (i) largely indirect and (ii) triggered by HIV neurotoxicity (Kaul et al., 2001; Ellis et al., 2007; Saylor et al., 2016). As previously described, the proinflammatory reaction in the brain may alter CNS functions (Hsiao et al., 2013; Houser and Tansey, 2017). Thus, neuroinflammation is now considered to be a key factor in the evolution of many neurodegenerative diseases (González et al., 2014; Houser and Tansey, 2017). To illustrate this, observations from recent studies using MRI combined with metabolite spectroscopy have confirmed the presence of persistent neuroinflammation in individuals with HAND (Cysique et al., 2018; Alakkas et al., 2019; McArthur and Johnson, 2020). McArthur and Johnson have also reported that even virologically suppressed HIV-infected individuals display sustained inflammation and neural injury. These investigators have further suggested that a reduction of neuroinflammation and systemic inflammation may protect the CNS from immune-mediated damage (McArthur and Johnson, 2020). In addition to the inflammation resulting from the presence of HIV within the brain, previous studies have also shown that microbial translocation may drive neuroinflammation and thereby contribute to the pathogenesis of HAND (Ancuta et al., 2008; Vera et al., 2015). Indeed, HIV infection leads to gut microbiota imbalance, increases intestinal permeability, and causes persistent release of microbial products into the bloodstream (Lackner et al., 2009). The preceding mechanisms, as observed in past research, significantly contribute to systemic inflammation (Mehraj et al., 2020; Luo et al., 2022). Interestingly, several published articles have demonstrated that probiotic supplementation may positively influence neuronal functions, decrease neuroinflammation, and ameliorate cognitive impairment in PLWH (; Ceccarelli et al., 2017). Additionally, multiple studies have shown that reduced gut microbial diversity and reduction of their associated metabolites contribute to the development of neuroinflammation and cognitive impairment (Magnusson et al., 2015; Beilharz et al., 2016; Li et al., 2019; Saiyasit et al., 2020). Thus, it can be assumed that HIV infection within the gut may indirectly provoke HIV-associated neuronal damage and neurodegenerative diseases in PLWH.

Patients with HIV-1 infection have been shown to have reduced serotonin levels in their blood and CSF (Launay et al., 1988; Keegan et al., 2016; Fu et al., 2023). Gut microbiota may regulate the synthesis of serotonin, and this may be related to an imbalance of gut microbiota composition and the alterations observed in levels of microbiota-associated metabolic products. As reported previously, factors related to the decrease in serotonin levels include a decrease in the abundance of symbiotic gut microorganisms which upregulate serotonin synthesis, an increase in the specific gut microbiota that regulate the kynurenine pathway, and a decrease in the microbial metabolites responsible for serotonin synthesis or release (O’Mahony et al., 2015; Gao et al., 2020; Agirman and Hsiao, 2021). However, it is possible that low levels of serotonin can be therapeutically enhanced, as researchers have demonstrated that specific probiotic supplementation may significantly increase serum serotonin levels in PLWH (Scheri et al., 2017). Serotonin is known as a key neurotransmitter which is involved in a wide range of mood, behavioral and cognitive functions (Cryan and Leonard, 2000; Berger et al., 2009). Low levels of serotonin have been associated with depression, fatigue, and impaired cognitive functions (Geldenhuys and Van der Schyf, 2011; Kaur et al., 2019). Interestingly, several past publications have shown that many HIV-positive patients with depression respond well to oral treatment with selective serotonin-reuptake inhibitors (SSRIs) (Caballero and Nahata, 2005). Accumulated evidence confirms that changes in gut microbiota composition and of their metabolites in PLWH may influence intrinsic serotonin production, and therefore promote the pathogenesis of depression and impaired cognitive functioning.

HIV infection may lead to the enrichment of gut-resident bacteria with the capacity to metabolize tryptophan (TRP) through the kynurenine (KYN) pathway, and these gut-resident bacteria may augment the production of kynurenine metabolites through increased expression of gut IDO1 (Rhee et al., 2005; Atarashi et al., 2011; Vujkovic-Cvijin et al., 2013). Several recent reports have shown that in HIV-infected individuals, serum TRP concentration is markedly decreased while the kynurenine concentration is increased, as reflected in elevated KYN to TRP (KYN/TRP) ratios (Huengsberg et al., 1998; Qi et al., 2018). Furthermore, it has been observed that severity of depression in HIV patients is associated with a decrease in plasma tryptophan concentration and an increase in the KYN/TRP ratio (Martinez et al., 2014). The TRP-KYN pathway may also lead to changes in downstream metabolites (Heyes et al., 1991; Qi et al., 2018). Some of these metabolites may be neurotoxic, such as 3-hydroxykynurenine (3-HK), 3-hydroxyanthranilic acid (3-HAA), and quinolinic acid (QUIN) (Stone, 1993; Guillemin et al., 2003, 2005; Davies et al., 2010; Capuron et al., 2011; Keegan et al., 2016). More importantly, fluctuating levels of kynurenine pathway metabolites have been observed to be associated with several neurologic and psychiatric disorders (Schwarcz et al., 2012; Kennedy et al., 2017). Indeed, cerebrospinal fluid (CSF) levels of quinolinic acid have been shown to increase during HIV infection, and to be associated with HAND severity (Heyes et al., 1989, 1991; Achim et al., 1993; Sei et al., 1995; Valle et al., 2004) Therefore, given the regulatory role of the gut microbiota on kynurenine pathway metabolites during HIV infection and the impact of kynurenine pathway metabolites on neurocognitive impairment, it is reasonable to suggest that HIV infection causes gut microbiota imbalance, promotes the activation of IDO, and favors the continuous accumulation of neurotoxic metabolites, which ultimately fosters the development of neurocognitive disorders.

**Figure 1:**
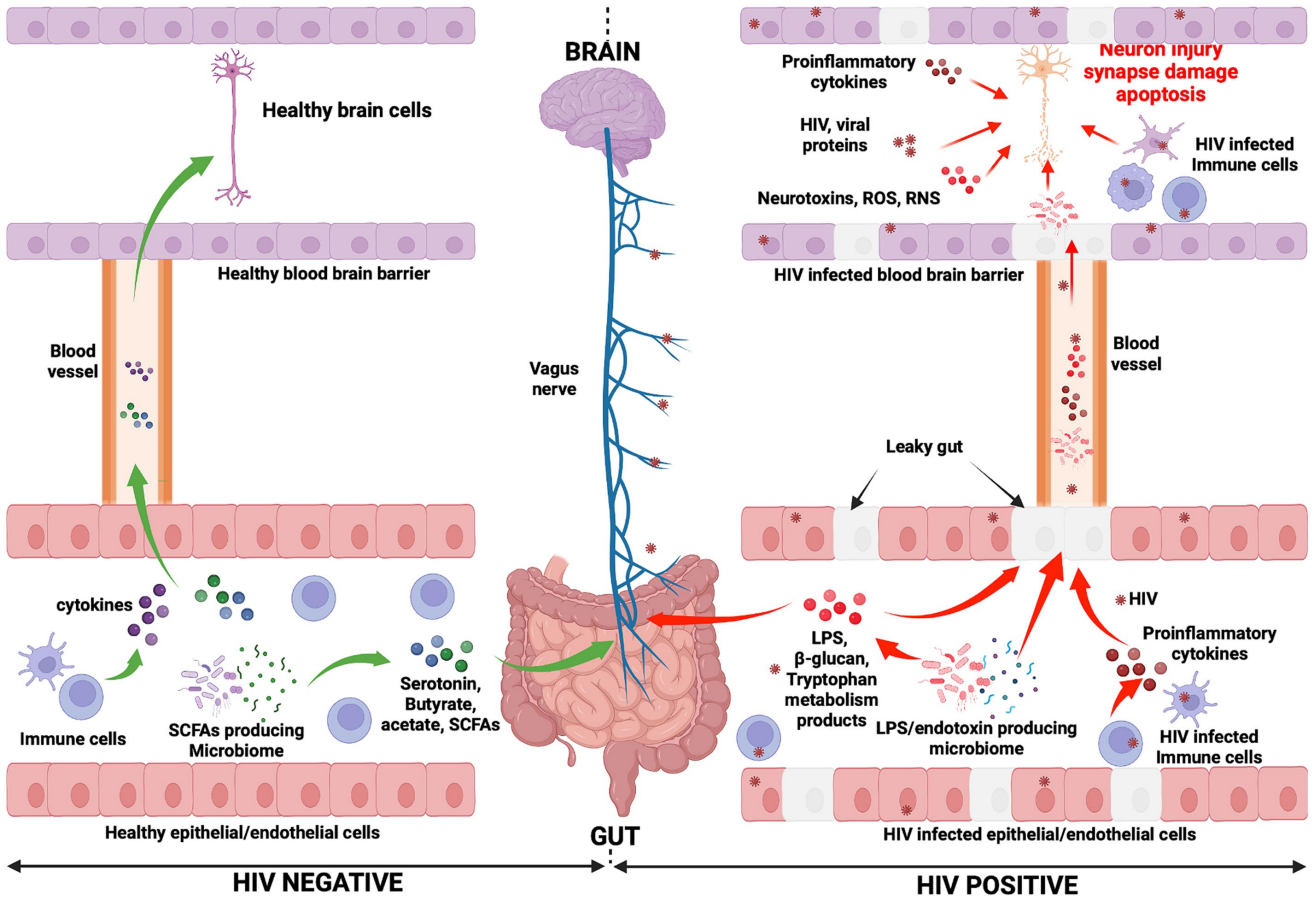
Mechanisms potentially influencing HAND manifestation. In the HIV negative context, immune cells produce cytokines and influence the functioning of the brain (negatively or positively depending on the presence of inflammation or not, respectively) (Haroon et al., 2014, 2016; Haroon and Miller, 2017). They reach the brain through the systemic circulation. Gut microbiota, particularly SCFA-producing microbiota, induce the production of neurotransmitters (serotonin) and metabolites (acetate, butyrate, and SCFAs). Consequently, the vagus nerve is stimulated by these molecules and transmits signals to the brain (Needham et al., 2020). Neurotransmitters and metabolites are able to cross the blood–brain barrier (via the systemic circulation) to subsequently directly affect brain health. Conversely, in the context of HIV infection, the gut epithelial/endothelial barrier and the blood brain barrier are disrupted and consequently are unable to selectively filter elements, which originate in the gut and consequently enter the brain. The production of different dysfunctional elements are also promoted. SCFA-producing microbiota are depleted and replaced by LPS/endotoxin-producing microbiota. The latter are potentially pathogenic microbes, which produce endotoxins. The reduction of SCFA-producing microbiota induces a reduction in the production of serotonin and other beneficial metabolites (butyrate, acetate), while the production of LPS and other endotoxins are promoted by LPS/endotoxin-producing microbiota. In the HIV infection context, immune cells are depleted and produce proinflammatory cytokines, which exert a negative influence on brain cell development and functioning. The preceding elements (LPS, endotoxins, and proinflammatory cytokines) cause abnormal functioning of the vagus nerve, which may induce a sluggish intestinal transit time (constipation) which further promotes the growth of pathogenic bacteria within the gut (George et al., 2014). Furthermore, the preceding toxic elements may then easily reach the brain through the systemic circulation. Within the brain, endotoxins, HIV-infected immune cells (microglia, astrocytes, and macrophages, which produce proinflammatory cytokines, neurotoxins, ROS, and RNS), specific pathogenic microbes (potentially less likely), and HIV viral proteins work together to further compromise neuronal integrity, resulting in neuronal damage, synapse destruction, and ultimately neuronal death (Navarathna et al., 2016; Zhu et al., 2020). The preceding events which occur during HIV infection may well be seen as the potential pathogenic mechanisms whereby the gut-brain axis induces HAND development. ROS, Reactive oxygen species; RNS, Reactive nitrogen species.

**Figure 2:**
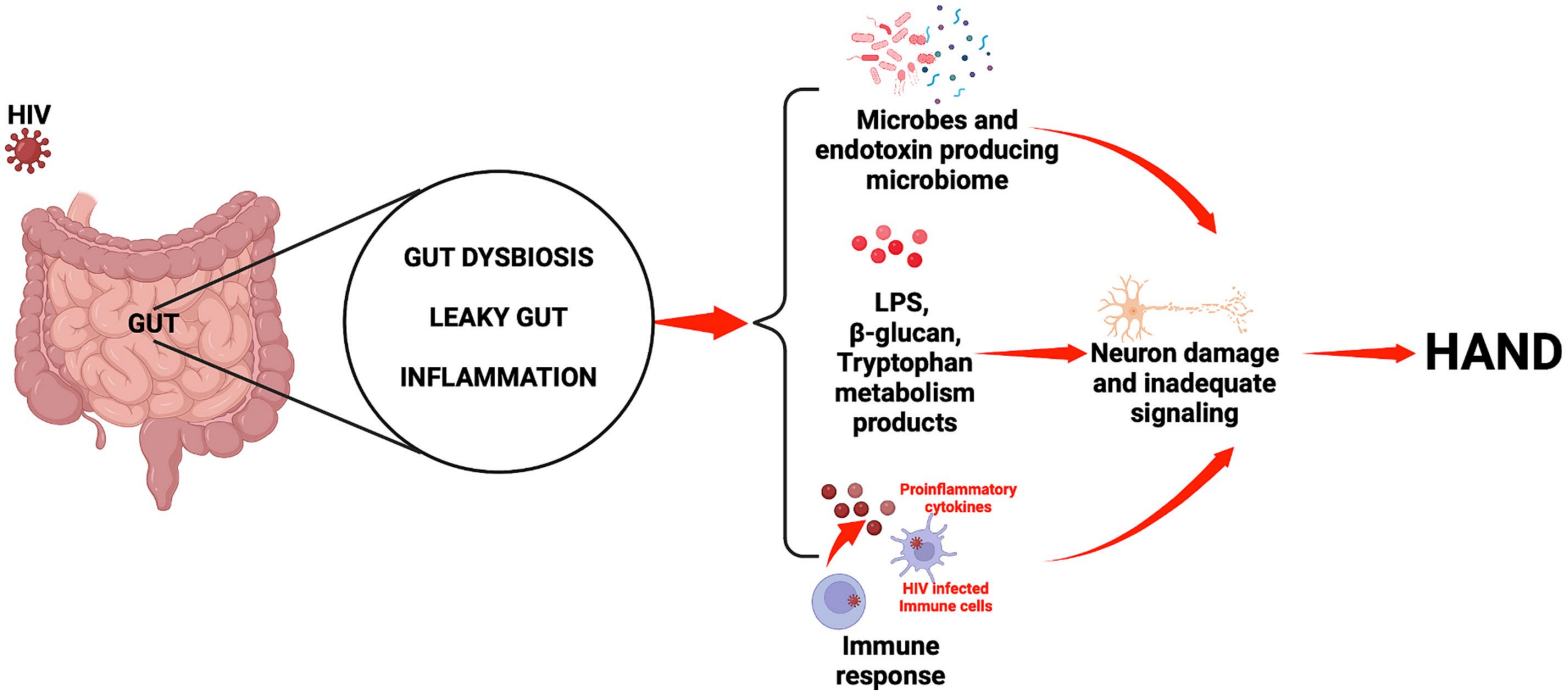
Relationship between HIV, the gut microbiota, and HAND.

An overall picture of the potential mechanisms whereby the gut-brain axis may influence HAND development during HIV infection is presented in Figure 1. Furthermore, an illustration of the relationship between HIV, gut microbiota, and HAND is provided in Figure 2.

## 6 Conclusion and perspectives

It is important to remember that HIV is a neurotropic virus which has the ability to affect the brain and to disrupt brain functions, and this may well account for the development of HAND. However, cumulative research evidence points, also, to the influential role of the microbiota-gut-brain axis in HAND development. Indeed, HIV-related gut associated dysbiosis syndrome, translocated microbial products, and sustained systemic inflammation are key features of HIV infection, which profoundly disrupts the homeostasis of both the gut and the brain. It is known that the gut and the brain interact via elements such as the autonomic nervous system, microbiota and their metabolites, the immune system, and the endocrine system. Herein, we have considered and discussed how any change that occurs in the composition or functions of the preceding elements as a consequence of HIV infection may significantly dysregulate homeostasis of the gut-brain axis, and thus encourage the pathogenesis of HAND. The information reported in this article represents nuggets of compelling evidence, which exposes the potentially fundamental role played by the microbiota-gut-brain axis in the development of HAND. We have shown that HAND may result from inflammation, HIV-associated gut dysbiosis syndrome, and the leaky gut. As known consequences of HIV infection, these processes induce profound alterations in microbial metabolite composition (SCFA, serotonin, and tryptophan) and favor immune responses which, cumulatively, result in neuronal damage and/or inadequate neuronal signaling (Figure 2).

Despite the preceding observations, further evidence is required to establish the precise role of the microbiota-gut-brain axis during HAND pathogenesis. As such, it is necessary to further explore the correlation between specific gut microbiota components and HAND in future research. Perhaps interventions using antibiotics, probiotics, and fecal microbiota transplantation (FMT) may (i) regulate composition and metabolites of gut microbiota, (ii) regulate the homeostasis of the microbiota-gut-brain axis, and (iii) thereby prevent or alleviate potential neurological diseases in PLWH. Apart from dietary interventions, the administration of cannabinoid based drugs [such as dronabinol, a synthetic version of delta-9-tetrahydrocannabinol (THC)] at low dose may (i) regulate the microbiome-gut-brain axis, (ii) reduce neuroinflammation, and (iii) mitigate HAND pathogenesis in the context of HIV infection, as has been demonstrated recently (McDew-White et al., 2023).

Despite this curated narrative review of the contemporary literature being focused on the relationship between HIV infection, gut microbiota dysbiosis, and HAND, the absence of original data represents a major limitation to this work. Nonetheless, we believe that our work may serve to pave the way toward robust future investigations (particularly clinical trials) in this compelling area of endeavor.

## Author contributions

AH: Writing – original draft. SZ: Writing – original draft. VH: Writing – review & editing. XW: Conceptualization, Writing – review & editing. JO: Conceptualization, Writing – review & editing. YC: Conceptualization, Writing – review & editing.
